# Satisfaction with childbirth services provided in public health facilities: results from a cross- sectional survey among postnatal women in Chhattisgarh, India

**DOI:** 10.1080/16549716.2017.1386932

**Published:** 2017-10-31

**Authors:** Paridhi Jha, Margareta Larsson, Kyllike Christensson, Agneta Skoog Svanberg

**Affiliations:** ^a^ Department of Women’s and Children’s Health, Uppsala University, Uppsala, Sweden; ^b^ Department of Women’s and Children’s Health, Karolinska Institutet, Stockholm, Sweden

**Keywords:** Hindi-translated Scale for Measuring Maternal Satisfaction, intrapartum care, institutional births, Caesarean section, quality of care

## Abstract

**Background**: A woman’s satisfaction with childbirth services can have a significant impact on her mental health and ability to bond with her neonate. Knowing postnatal women’s opinions and satisfaction with services makes the services more women-friendly. Indian women’s satisfaction with childbirth services has been explored qualitatively, or by using non-standard local questionnaires, but scientific data gathered with standardised questionnaires are extremely limited.

**Objective**: To measure postnatal Indian women’s satisfaction with childbirth services at selected public health facilities in Chhattisgarh, India.

**Methods**: Cross-sectional survey using consecutive sampling (*n *= 1004) was conducted from March to May 2015. Hindi-translated and validated versions of the Scale for Measuring Maternal Satisfaction for Vaginal Births (VB) and Caesarean Births (CB) were used for data collection.

**Results**: Although most of the women (VB 68.7%; CB 79.2%) were satisfied with the overall childbirth services received, those who had VB were least satisfied with the processes around meeting their neonates (mean subscale score 1.8, SD 1.3), while women having CB were least satisfied with postpartum care received (mean subscale score 2.7, SD 1.2). Regression analyses revealed that among women having VB, interacting with care providers, being able to maintain privacy, and being free from fear of childbirth had a positive influence on overall satisfaction with the childbirth. Among women having CB, earning their own salary and having a positive perception of self-health had associations with overall birth satisfaction.

**Conclusions**: Improving interpersonal interaction with nurse-midwives, and ensuring privacy during childbirth and hospital stay, are recommended first steps to improve women’s childbirth satisfaction, until the supply gap is eliminated.

## Background

The complex and multidimensional nature of satisfaction with received health services is well established [1–3], and measuring beneficiaries’ satisfaction with health services, including childbirth services, has emerged as a universal cost-effective method of evaluating service quality [4,5]. Measuring women’s satisfaction with childbirth services not only helps in improving client-friendliness and cultural sensitivity of facility-based intrapartum and postpartum care [6]; it also has clinical significance. Studies show that women who are satisfied with childbirth services tend to have better self-esteem and confidence, are faster in establishing a maternal–neonatal bond, and are more likely to breastfeed compared with women who are dissatisfied [7,8]. Women who are dissatisfied with their childbirth experiences are more prone to develop a fear of childbirth and postnatal depressive symptoms, and to face difficulties in breastfeeding and in performing baby and self-care [9,10].

Several factors influence women’s satisfaction with childbirth services: certain demographic characteristics have been predominantly studied – without a global consensus – in relation to satisfaction with childbirth services. For example, a Swedish study (*n *= 2762) reports that younger women had more negative expectations related to childbirth and they experienced more pain and lack of control during labour compared with older women [11], while a Brazilian survey (*n *= 15,688) showed no age-related difference in women’s satisfaction with childbirth services [12]. Studies from low- and middle-income countries show that satisfaction with services had a negative association with the amount of time women spent at the health facility before childbirth [5,13,14]. The educational level of women – in different studies and settings – has demonstrated positive, negative or nil association with satisfaction with childbirth services [4,12,15]. Working women tend to have lower satisfaction levels with childbirth services compared with homemakers [16]. Having a fear of childbirth and/or postnatal depressive symptoms has a negative and compounding association with satisfaction [17–19].

Other identified factors that influenced satisfaction with childbirth services are: having clean and orderly labour rooms and women-friendly childbirth processes, such as having been prepared in advance for what to expect during the labour/postpartum/breastfeeding period; involvement in the decision-making process; having a birth plan and being able to follow it; having pain relief during labour; having a birth companion and respectful care providers; receiving help from care providers in performing self and neonate’s care; and experiencing less symptoms in the postpartum period [3–5,15,20–27]. A woman’s obstetric history, mode of childbirth, and her feelings towards recent childbirth can also affect childbirth satisfaction. For example: being multiparous, preferring a spontaneous vaginal birth and being able to have a spontaneous vaginal birth [15,28] enhances the women’s satisfaction with giving birth. The feelings associated with childbirth itself, due to limited opportunities of exploration in quantitative studies, pose some confounders like the ‘halo effect’ – a positive attitude prevailing due to successfully giving birth – making it difficult to separate childbirth satisfaction from satisfaction with childbirth services [12,29,30]. The ‘ceiling effect’ – participants’ tendencies to rate services more positively in general – is another known confounder, making it difficult to differentiate excellent care from adequate care [23,31]. Participants’ subjectivity – being pleased with services that are not necessarily evidence-based [32–34] – poses another confounder for quantitative studies measuring satisfaction. While qualitative studies have emerged as an alternative to study satisfaction, they involve a small group of participants who have been purposefully selected, and findings from such studies rarely can be generalised for programmatic changes, especially so in big countries, like India.

Like other low- and middle-income countries, the quality of childbirth services in Indian public health facilities is receiving growing attention. The multi-front promotion of institutional childbirths by the Government of India has resulted in an exponentially increasing number of women coming to hospitals with the chief expectation of ensuring the safety of themselves and their neonates [35–38]. However, the expansion in the number of labour rooms and necessary resources and infrastructure, especially in public health facilities, has not been proportional to rising demand [36,39,40]. Labour rooms have become crowded and understaffed and face shortages of equipment and material [17,40] and the documentation processes around childbirth have been described to be ‘too much to do concurrently with actual care provision’, with care providers relying on verbal communication rather than maintaining written documentation of decision-making around childbirth [17,41]. Qualitative studies exploring Indian women’s experiences and opinions on giving birth at a health facility reveal that they are not fully satisfied with their childbirths, primarily due to the long waiting time before they meet a healthcare provider, having few opportunities to communicate with providers, not being involved in decision-making, and having stern care providers [17,35,42,43]; however, they settle for childbirth services perceived as ‘essential’ for safe childbirth rather than ‘desirable’ for a pleasant experience [17,44,45].

While the community’s access to institutional childbirths has improved, the assumption that accessibility is synonymous with quality of care, especially among policymakers, gives concern. India and Chhattisgarh, in this context, are similar to the trends observed in other lower- and middle-income countries [46].

To the best of our knowledge, no study in India has used a standardised scale to measure women’s satisfaction with childbirth services, making it difficult to compare Indian findings with results from other countries that used the same tool. This also potentially creates a barrier for cross-cultural learning. The present study aimed to measure Indian women’s satisfaction with institutional childbirths using a standardised scale in two districts of Chhattisgarh State, India, with an intent to potentially use the findings in advocacy for service improvement.

## Methods

### Study design and participants

A cross-sectional survey in the postnatal wards of 17 public health facilities (two District Hospitals and 15 Community Health Centres [CHCs]) in two districts of Chhattisgarh State, India, was conducted to measure women’s satisfaction with childbirth services. All women (*n *= 1004) who had had an uncomplicated vaginal birth as defined by WHO criteria [47], were invited to participate. Women who had a caesarean birth (planned or emergency, where the mother and neonate proceeded to an uneventful postnatal period) were included in the study. Another inclusion criterion was giving birth to healthy single neonate in one of the selected health facilities. Participants who were on the ‘do not disturb’ list by treating obstetricians’ orders were excluded. Women whose neonates were admitted to intensive care for any reason were excluded to minimise confounders.

### Study setting

Childbirth services in India are primarily provided to women in two levels of health facility. The CHCs are 30- to 60-bedded health facilities that serve as secondary-level health care and are designed to provide specialist care to the rural and sub-urban population [48]. The CHCs should have a health team consisting of 12 specialists/general physicians, and 11 nurse-midwives and public health nurses. All CHCs offer services for vaginal childbirths; some also offer caesarean section services [48].

The District Hospitals (DHs) are 101- to 500-bedded health facilities that provide secondary-level healthcare services while serving as the secondary referral units within a given geographic region. In addition, the DHs should be sensitive and responsive to complicated cases and emergency referrals from the CHCs. All DHs provide caesarean section services in addition to vaginal birth facilities [49]. A 300-bedded DH health team should have 50 specialists/general physicians, and 135 staff nurse-midwives [49].

In public health facilities, nurse-midwives are the primary care providers for women having vaginal births, whereas obstetricians/physicians are the primary care providers for the women who have caesarean births and supervise the care of women who have complicated vaginal births. *Mitanins* (village-level non-technical change agents trained to promote institutional births as a means to reduce maternal and neonatal mortality) encourage women to have institutional births, and accompany them to public health facilities for childbirths. The public healthcare system in Chhattisgarh state suffers with acute shortages of manpower, with nearly 70% of nurse-midwife posts remaining vacant. This shortage is also acute among physicians/specialists and paramedical staff in Chhattisgarh [50,51],In this study, 15 CHCs out of 22 total available CHCs (having 10 or more childbirths per month), and two DHs (both districts have one DH each) having more than 20 childbirths per day, were selected for data collection. Demographic data and past obstetric history – current age, age at marriage, duration of married life, education, social category, perception of self-health, gravidity, parity, miscarriages, number of living children – were collected from participants at the start of data collection. Some data – maintenance of partograph, duration of hospital stay before childbirth, completed weeks of gestation, mode of childbirth, designation of primary care provider, neonate’s birthweight – were obtained from participants’ medical records. While the CHCs and DHs had instruments available for assisted vaginal births (vacuum and forceps), no assisted births were performed during the period of data collection. In case of caesarean births, the records documented the final mode of childbirth with operation notes. Many times, the files lacked a clear indication for performing caesarean section. Also, it was unclear from the medical records if the caesarean section performed was planned or not.

### Sampling

A consecutive non-random sampling method was used to recruit participants and written informed consent was taken from all who agreed to participate. Power analysis using PASS 14 software was carried out to determine the required sample size. In this study, the significance level (α) for *p*-values was set at 5% in a two-tailed test; type 2 error (β) at 80%; and confidence intervals (CI) at 95% [52]. This calculation provided the minimum required sample size (*n *= 815) that was to be recruited to generate adequately sized subgroups to ensure the statistical robustness of analyses. Considering the number of refusals and mid-interview dropouts recorded in the pilot studies (33%), the sample size was fixed at 1200 respondents.

### Data collection

An eight-member team consisting of the first author and seven research assistants, who had previous experience of conducting surveys, conducted one-to-one interviews with recruited participants at their bedside on the second or third day after vaginal birth, and on the fourth or fifth day after caesarean birth. Individual interviews for all participants ensured that both literate and illiterate women could participate. The data collection schedule was developed with input from the hospital staff so that ward rounds, procedures and treatment, and family meeting times were not disturbed. Data collection commenced in the available time pockets from 7.00 am to 7.00 pm for 42 consecutive days, from March to May 2015.

### Questionnaire

The questionnaire consisted of three parts: (1) open-ended questions on demographic information; (2) a ‘Yes/No’ questionnaire on the basic facilities that the respondents were able to procure in a public health facility (own bed, bedsheets, pillows, personal lockers, interaction frequency with providers) – some selected through review of literature, others developed based on findings from a qualitative study [44] done in the same settings, exploring women’s experiences of having institutional births; and (3) Hindi-translated and validated in India versions of the Wijma Delivery Experience Questionnaire Version B (WDEQ-B); Edinburgh Postnatal Depression Scale (EPDS); and Scale for Measuring Maternal Satisfaction (SMMS): Normal (Vaginal) and Caesarean Births [53–55].

The Hindi WDEQ-B is a 33-item Likert scale with a score range of 0–165. Scores above 85 indicate severe (more than normal) fear of childbirth, whereas scores above 99.5 indicate clinical fear of childbirth (interfering with the woman’s activities of daily living). Scores below the cut-off indicate absence of severe/clinical fear in the respondent [53]. The Hindi EPDS is a 10-item Likert scale with a possible score range of 0–30. Scores of 10 and above indicate postnatal depressive symptoms, and a score of 12 and above shows probability of a psychiatric illness requiring clinical attention [54]. The Hindi-translated SMMS scales (Normal and Caesarean Births) are 36-item Likert scales with a possible total score range of 36–180. The cut-off scores – the response score that distinguishes women in to two groups: more satisfied, and less satisfied, with received childbirth care – for the Hindi-translated SMMS Normal and Caesarean Birth are 105.5 and 108.5, respectively [55].

### Data analysis

Richard Baker’s Pragmatic Model of Patient Satisfaction in General Practice was used for a comprehensive interpretation and presentation of findings [31]. The pragmatic model allows researchers to measure satisfaction with health services using only the available data, thus avoiding the recourse to socio-psychological theories of behaviour [31]. The key components of Baker’s Pragmatic Model are: (1) patient characteristics; (2) communicated requirements; (3) patients’ prioritisations for personalised care; (4) elements of care provided; (5) reaction to the care received; (6) interaction with health care; and (7) level of satisfaction.

Before applying the model; data were processed using IBM software SPSS 24. First, only those questionnaires where the participant had answered all items on the satisfaction scales were included in data analyses. Groups for each demographic variable were distinguished by categorising women with similar characteristics, which allowed a comprehensive demographic description of participants. For example: two categories of age were formed with median age as the cut-off, as there were very few participants below 20 or above 30 years old. Women were also grouped based on their social categories, as described in the constitution of the Republic of India (Scheduled Castes [SC], Scheduled Tribes [ST], Other Backward Classes [OBC] and General [G] category), occupation (homemakers, earning a salary), perception of self-health (positive, negative), highest level of education received (never attended formal school, passed middle school, completed formal schooling, went to/going to college), total pregnancies (primigravida, multigravida), parity (primipara, multipara), history of spontaneous abortions (yes, no), current gestation length (preterm, term, post-term), facility chosen for childbirth (DH, CHC), time spent in health facility before childbirth (up to six hours, more than six hours), mode of childbirth (vaginal birth, caesarean birth), opportunity to interact with care providers (less than once a day, at least once a day), sex of the neonate (female, male), and birthweight of the neonate based on Indian reference baby guidelines, Government of India (<2500 g, ≥2500 g). Women were divided into two groups based on their responses of having received basic facilities in a healthcare facility (yes, no).

For a clear empirical presentation of scores obtained, the response scores on Hindi-translated SMMS, WDEQ-B and EPDS scales were divided into two groups using cut-off scores (less satisfied/more satisfied; not having/having fear of childbirth; not having/having depressive symptoms). For satisfaction scales, the item-wise average score for the subscales (sum of individual scores for each item in subscale/number of items in subscale) was calculated for each participant. Calculating the average of all participants’ subscale scores revealed the childbirth service areas with least and highest item-wise average scores. Factors associated with fear of childbirth and depressive symptoms among the same participants have been published separately [53].

Statistical calculations involved non-parametric two-tailed χ^2^ tests to measure the level of associations that the influencing factors had with women’s satisfaction scores; odds ratio (95% CI) to compare chances of receiving basic facilities (women who had caesarean birth versus women who had vaginal births), and separate linear regression models for vaginal births and caesarean births with those variables that had significant association (χ^2^ tests) with satisfaction scores. Data from some variables – age, duration of marriage, age at marriage, scores of standard scales mentioned above, birthweight of the neonate, time spent in health facility before childbirth – were fed to the linear regression model as continuous data to rule out ‘categorisation’ bias.

### Results

Out of 1301 women who gave birth at selected health facilities during data collection, 84 were excluded at the onset due to having stillbirths or having physicians’ orders not to be disturbed. A further 85 women refused to participate when approached, either upon not having family’s permission to engage in survey (76.5%) or due to not feeling up to answering the questionnaire. A total of 1131 women completed the survey (93% response rate) and, on average, each interview took 23 minutes to complete. 127 questionnaires were later excluded from the analyses due to being incompletely answered.

**Figure 1.**
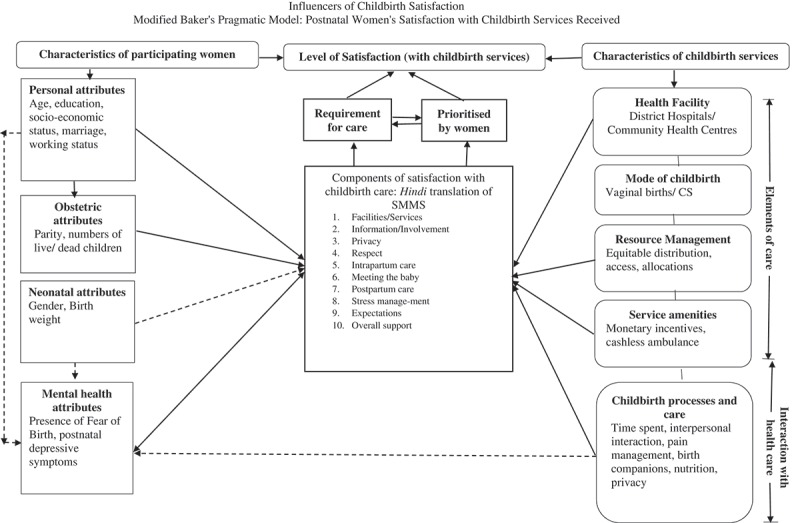
Influencers of childbirth satisfaction modified Baker’s Pragmatic Model: postnatal women’s satisfaction with childbirth services received.

A modified pragmatic model of Women’s satisfaction with childbirth services received was developed based on the findings (Figure 1). In the modified model, the top right and left boxes present the two overarching systems – characteristics of participating women, and characteristics of childbirth services – that conceptually affect levels of satisfaction (top middle box, Figure 1). The components of satisfaction with childbirth services – as identified in the Hindi questionnaires’ psychometric assessment [55] and displayed in the central broad-margined box – demonstrate a state of equilibrium between what women require from a good institutional birth and what they prioritise. This can sometimes be interchangeable, hence demonstrated by the bi-directional arrows. The balance between the women’s requirements and prioritisation affects the overall level of satisfaction with childbirth services.

The characteristics of women (the series of boxes on the left-side of the model) can be further grouped as personal, obstetrics, neonate-related and women’s mental-health-related attributes. Associations among these attributes and components of satisfaction as found in this study are shown by unidirectional solid arrows.

The health care characteristics – presented in the soft-edged boxes on the right side – are conceptually divided into two groups. The ‘elements of care’, comprising of health facility, mode of childbirth, resource management at health facility, and service amenities provided. ‘Interactions with health care’ comprises OF all processes and childbirth practices such as time from admission to childbirth, interpersonal interactions, pain management during childbirth, having birth companions, and nutrition and hydration during labour. The associations that were discovered in the same sample, published separately [53], are shown as dashed arrows. Detailed descriptions of the findings are as follows:

#### *Description of respondents (* Table 1)

The mean age of respondents was 23.7 years (SD 3.4). The majority (80.4%) had come to the public health facility for childbirth after being encouraged by the *Mitanins*. Grandmultiparity (having five or more children) was observed in <6% of the respondents. Only 8% participants had a completed partograph attached to their medical records.

**Table 1.** Demographic characteristics of the respondents.

	Caesarean births, *n* (%)	Vaginal births, *n* (%)		
	DHs (*n* = 144)	DHs (*n* = 499)	CHCs (*n* = 361)	Total
Age in years, mean (SD) (*n* = 1004)	23.9 (3.6)	23.5 (3.5)	23.7 (3.4)	23.7 (3.5)
Duration of married life in years, mean (SD) (*n* = 1004)	3.76 (2.6)	3.9 (3.5)	4.3 (4.1)	4.8 (3.4)
Age in years at marriage, mean (SD) (*n* = 1004)	20.9 (3.1)	19.4 (2.2)	19.4 (1.9)	19.5 (2.3)
Social category (*n* = 982)				
Scheduled Caste (SC)	29) (20.7)	79 (16.2)	36 (11.6)	144 (14.7)
Scheduled Tribe (ST)	13 (9.3)	62 (12.8)	80 (25.7)	155 (15.8)
Other Backward Classes (OBC)	89 (63.7)	298 (61.3)	195 (62.7)	582 (59.3)
General (G)	9 (6.3)	47 (9.7)	45 (14.5)	101 (10.2)
Highest level of education (*n* = 989)				
Never been to school	8 (5.6)	40 (8.1)	28 (7.8)	76 (7.7)
Up to 8 years of formal school	67 (48.3)	209 (42.4)	202 (56.6)	478 (48.3)
9–12 years of formal school	47 (33.8)	211 (42.8)	115 (32.2)	373 (37.7)
Doing graduation/graduate or above	17 (12.3)	33 (6.7)	12 (3.4)	62 (6.3)
Working status (*n* = 1000)				
Homemaker	81 (56.2)	334 (68.0)	280 (76.7)	695 (69.5)
Earning own salary	63 (43.8)	157 (32.0)	85 (8.5) (23.3)	305 (30.5)
Perception of self-health (*n* = 993)				
Very good/Good	123 (85.4)	415 (85.6)	328 (89.0)	866 (87.2)
Bad/Very bad	21 (14.6)	70 (14.4)	36 (11.0)	127 (12.8)
Total numbers of pregnancies experienced (*n* = 1004)				
Primigravidae	68 (47.2)	236 (48.0)	144 (39.1)	448 (44.6)
Multigravidae	76 (52.8)	256 (52.0)	224 (60.9)	556 (55.4)
Parity (*n* = 1004)				
Primiparous	72 (50.0)	240 (48.1)	156 (43.2)	468 (46.6)
Multiparous	72 (50.0)	259 (51.9)	205 (56.8)	536 (53.4)
Had previous spontaneous abortions (*n* = 1004)				
Never	138 (95.8)	485 (97.2)	326 (90.3)	949 (94.5)
At least once	6 (4.2)	14 (2.8)	35 (9.7)	55 (5.5)
Time spent in the health facility from admission till childbirth (*n* = 999)				
≤6 hours	51 (35.4)	324 (65.1)	268 (75.0)	643 (64.4)
>6 hours	93 (63.6)	174 (34.9)	89 (25.0)	356 (35.6)
Primary care provider during labour and childbirth (*n* = 995)				
Obstetrician/Physician	144 (100.0)	14 (2.9)	6 (1.6)	164 (16.5)
Nurse-Midwives/ANMs	0 (0.0)	474 (97.1)	357 98.4)	831 (83.5)
Received perineal suturing for tear/episiotomy (*n* = 850)				
Yes	NA*	206 (41.9)	143 (39.9)	349 (41.1)
No	NA	286 (58.1)	215 (40.1)	501 (58.9)
Received local anaesthesia before perineal wound suturing (*n* = 349)				
Yes	NA	63 (30.7)	81 (56.2)	144 (41.3)
No	NA	142 (59.3)	63 (43.8)	205 (58.7)
Sex of the neonate (*n* = 1004)				
Female	74 (51.4)	237 (47.5)	174 (48.2)	485 (48.3)
Male	70 (48.6)	262 (52.5)	187 (51.8)	519 (51.7)
Birthweight of the neonate (*n* = 963)				
≤1500 g	3 (2.1)	24 (5.1)	1 (0.3)	28 (2.9)
1501–2499 g	22 (15.5)	67 (14.2)	62 (17.8)	151 (15.7)
≥2500 g	117 (82.4)	381 (80.7)	286 (81.9)	784 (81.4)
Received monetary incentive for institutional births (*n* = 1004)				
Yes/Will receive	115 (79.9)	373 (74.7)	335 (92.8)	823 (81.9)
No/Don’t know	29 (20.1)	126 (25.3)	26 (7.2)	181 (18.1)
Presence of fear of childbirth (n = 1004)				
No	31 (21.5)	481 (96.4)	354 (98.1)	866 (86.3)
Yes	113 (78.5)	18 (3.6)	7 (1.9)	138 (13.7)
Presence of depressive symptoms (*n* = 1004)				
No	93 (64.5)	387 (77.6)	346 (95.8)	826 (82.3)
Yes	51 (35.5)	112 (32.4)	15 (4.2)	178 (17.7)

ANM, Auxiliary Nurse and Midwife; CHC, Community Health Centre; DH, District Hospital; NA = Not applicable.

#### *Description of basic services provided (* Table 2)

During labour and the initial postnatal period, most of the participants (84.3%) were left undressed from the waist down. All participants initiated breastfeeding within two hours of childbirth; however, 57% of the participants had no privacy during breastfeeding. Analyses of data from women giving birth in DHs showed that having a caesarean birth improved women’s likelihood of accessing certain basic facilities. For example, women who had caesarean births were four times more likely to have interpersonal interactions with care providers every day of their visit (*p *< 0.001) compared with women who had given vaginal births at the DHs. However, a separate analysis of data from all the women who had vaginal births – either at the DH or the CHC – revealed that women giving vaginal birth at the CHCs had better odds of receiving basic facilities compared to women who had vaginal births at the DHs. For example: women giving vaginal births at CHCs were twice as likely to have a bedsheet to cover themselves in labour rooms, compared with women at DHs (*p *< 0.001).

**Table 2.** Odds of receiving basic facilities among participants.

	Odds of receiving services upon having CB^A^ (*N* = 643)			Odds of receiving services upon having vaginal births at CHCs^B^ (*N* = 860)			
Basic services that are in common for care provision to both modes of childbirth (VB and CB)	OR^c^	CI (95%)	p value	OR^c^	CI (95%)	p value	Basic services – common and exclusive to normal births – being provided at CHCs and DHs
Got own bed	1.16	0.66–2.05	NS	1.16	0.58–2.33	NS	Got own bed
Got bedsheet from hospital to cover bed	3.74	2.77–5.06	<0.001	1.51	1.29–1.76	<0.001	Got bedsheet from hospital to cover bed
Got a locker to keep her belongings in ward	2.38	1.80–3.17	<0.001	1.36	1.16–1.59	<0.001	Got a locker to keep her belongings in ward
Got a bedsheet to cover herself in the ward	4.49	3.54–5.68	<0.001	2.0	1.73–2.31	<0.001	Got a bedsheet to cover herself in the ward
Got bedsheet to cover herself inside LR/Prep room	11.06	8.11–15.09	<0.05	1.68	1.36–2.06	<0.001	Got bedsheet to cover herself inside LR
Got sanitary pad from the hospital to use during early labour	2.87	1.39–5.89	<0.05	1.84	1.48–2.28	<0.001	Got sanitary pad from the hospital to use during early labour
Got sanitary pad from the hospital to use after childbirth	10.07	7.68–13.22	<0.001	2.0	1.62–2.44	<0.001	Got sanitary pad from the hospital to use after childbirth
Got privacy while breastfeeding her newborn	1.23	0.91–1.66	0.178	2.33	1.96–2.77	<0.001	Got privacy while breastfeeding her newborn
Had one or more interactions per day with nurse-midwife to clear doubts/get information	3.99	2.67–5.69	<0.001	5.98	3.99–8.97	<0.001	Had one or more interactions per day with nurse-midwife to clear doubts/get information
				0.413	0.35–0.48	<0.001	Received pillows to support on inside LR
				1.41	1.20–1.65	< 0.001	Got water to drink upon request
				1.59	1.36–1.86	<0.001	Simple food offered to women in early stages
				0.955	0.81–1.12	Ns	Had perineal suturing for perineal wounds
				1.83	1.42–2.35	<0.001	Received LA before perineal wound repair

CB, caesarean birth; CHC, Community Health Centre; CI, confidence interval; DH, District Hospital; LA, local anaesthesia; LR, labour room; OR, odds ratio; VB, vaginal birth.

^a^Calculated from responses of all women giving birth in DH, either VB (499) or CB (144).

^b^Calculated from responses of all women giving birth at either CHCs (361) or DH (499).

^c^Women who had vaginal births at the district hospital taken as reference in both scenarios mentioned above. Odds of receiving care: 1

#### *Women’s satisfaction with receiving perinatal and immediate postpartum services (* Table 3)

The mean score for SMMS–Normal Births was 115.25 (SD 17.8, range 63–155). However, the mean SMMS score of women giving birth at CHCs was 121.02 (SD 16.3, range 67–155) – higher than their counterparts at the DHs, whose mean score on SMMS was 111.4 (SD 17.8, range 63–152). Most of hat the participants having vaginal births (68.7%) were satisfied with the childbirth services they received; however, there was a significant difference in the proportion of women satisfied with childbirth services offered at CHCs (~81% satisfied) compared with women who gave birth at the DH (59.7% satisfied). Analysis of the subscales showed that women giving birth at the CHCs were most satisfied with ‘Maintaining privacy’ (mean score 4.7, SD 0.7), and with their overall ‘Experience of having an institutional childbirth’ (mean score 4.8, SD 0.5). Women having vaginal births at the CHCs were least satisfied with services pertaining to ‘Meeting the baby’ (mean score 1.4, SD 0.6).

**Table 3.** Participants’ scores for Hindi- translated SMMS–Normal Births and Hindi-translated SMMS–Caesarean Births; and prevalence of being satisfied with childbirth services.

	Satisfaction scores (SMMS–Normal Births) presented for respondents from CHCs (*n* = 361); DH (*n* = 499) and Overall (*n* = 860)^a^			Satisfaction scores (SMMS–Caesarean (*n* = 144)^b^
	CHC	DH	Overall	CB overall
Overall mean score (SD, range)	121.02 (16.34, 67–155)	111.14 (17.8, 63–152)	115.25 (17.8, 63–155)	123.94 (16.46, 78–153)
Overall satisfaction with services among respondents, n (%)				
	**CHC**	**DH**	**Overall**	**CB**
More satisfied	292 (80.9)	298 (59.7)	590 (68.7)	114 (79.2)
Less satisfied	69 (19.1)	201 (40.3)	269 (31.3)	30 (20.8)
*Mean scores (SD) for scale subgroups*				
Subscales	**CHC**	**DH**	**Overall**	**CB**
Facilities and services	3.5 (0.9)	3.2 (1.0)	3.3 (1.0)	3.6 (0.6)
Information and involvement in decision making	3.1 (1.0)	3.1 (0.9)	3.1 (1.0)	4.1 (0.7)
Maintenance of privacy**^d^**	4.7 (0.7)	1.3 (0.4)	3.0 (0.8)	**4.2 (0.5)**
Compassion and respect**^d^**	3.6 (0.7)	2.9 (1.0)	3.2 (1.0)	
Managing stress^c^	–	–	–	3.1 (1.2)
Intrapartum care received	3.8 (0.7)	3.7 (0.7)	3.7 (0.7)	3.6 (0.9)
Meeting the baby	**1.4 (0.6)**	2.1 (1.6)	**1.8 (1.3)**	2.8 (1.8)
Postpartum care received	3.0 (1.0)	1.8 (0.7)	2.4 (1.1)	**2.7 (1.2)**
Overall support provided	2.7 (0.8)	2.8 (0.7)	2.7 (0.8)	2.8 (0.6)
Expectations from institutional birth	3.7 (0.8)	2.7 (0.9)	3.2 (0.9)	3.0 (1.0)
**Experience of having institutional childbirth**	**4.8 (0.5)**	3.2 (0.6)	**4.0 (0.6)**	2.8 (0.5)

CB, caesarean birth; CHC, Community Health Centre; DH, District Hospital; SD, standard deviation; SMMS, Scale for Measuring Maternal Satisfaction.

^a^Scores >105.5 indicate higher satisfaction.

^b^Scores >108.5 indicate higher satisfaction.

^c^‘Managing stress’ is not a subscale in SMMS–Normal Birth.

^d^‘Having privacy, compassion and respect’ is a joint subscale in SMMS–Caesarean Birth.

Women having vaginal births at DHs scored lesser on all subscales compared with their CHC counterparts: these respondents were most satisfied with the ‘Intrapartum care’ they received (mean score 3.7, SD 0.7), and were least satisfied with ‘Maintaining privacy’ (mean score 1.3, SD 0.4).

For women who underwent caesarean section, the mean satisfaction score was 123.94 (SD 16.5, range 78–153) and 79.2% of the women were satisfied with the childbirth services they received. Analysis of the subscales showed that the subscales ‘Postpartum care received’ had the lowest mean scores (2.7, SD 1.2), whereas the subscale ‘Privacy, compassion and respect’ had the highest mean score (4.2, SD 0.5).

#### *Factors influencing childbirth satisfaction (* Table 4)

In both groups, higher levels of childbirth satisfaction were observed among women who: (1) were >22 years old (VB *p *< 0.05; CB *p *< 0.001); (2) had a positive perception of health (VB *p *< 0.05; CB *p *= < 0.001); (3) were earning their own salaries (VB *p *< 0.05; CB *p *< 0.001); and (4) did not have depressive symptoms (VB *p *< 0.05; CB *p *< 0.05).

**Table 4.** Association of demographic factors and access to basic facilities with overall satisfaction scores (vaginal and caesarean births).

	Vaginal births			Caesarean births		
	Not satisfied (SMMS score <105.5)	Satisfied (SMMS score >105.5)	*p*-value	Not satisfied (SMMS score <108.5)	Satisfied (SMMS score >108.5)	*p*-value
**Demographic variables**						
Age in years (VB: *n* = 857, CB: *n* = 143)			0.036			0.000
≤22 years	62 (23.4)	100 (17.7)		1 (3.3)	56 (49.6)	
>22 years	207 (76.6)	488 (82.3)		29 (96.7)	57 (50.4)	
Age at the time of marriage (VB: *n* = 858, CB: *n* = 143)			0.083			0.280
≤20 years	197 (73.5)	464 (78.6)		17 (56.7)	76 (67.3)	
≥21 years	71 (26.9)	126 (21.4)		13 43.3)	37 (32.7)	
Duration of married life in years (VB: *n* = 858, CB: *n* = 144)			0.237			0.958
≤2 years	125 (46.6)	237 (40.2)		12 (40.0)	45 (39.5)	
>2 years	143 (53.4)	353 (59.8)		18 (60.0)	69 (60.5)	
Highest level of education (VB: *n* = 859, CB: *n* = 142)			0.231			0.740
Never been to school	22 (8.2)	49 (8.3)		1 (3.4)	8 (7.2)	
≤8 years of formal education	119 (44.2)	296 (50.2)		14 (46.7)	54 (48.2)	
9–12 years of formal education	116 (43.1)	212 (35.9)		10 (33.4)	38 (33.9)	
Undergraduate/graduate/above	12 (4.5)	33 (5.6)		5 (16.7)	12 (10.7)	
Social category (VB: *n* = 851, CB: *n* = 140)			0.254			0.638
Scheduled Caste (SC)	42 (15.9)	76 (12.9)		6 (20.0)	22 (20.0)	
Scheduled Tribe (ST)	41 (15.5)	103 (17.6)		2 (1.4) (6.7)	10 (9.1)	
Other Backward Classes (OBC)	146 (55.3)	350 (59.6)		18 (60.0)	71 (64.5)	
General (G)	35 (13.3)	58 (9.9)		4 (13.3)	7 (6.4)	
Perception of self-health (VB: *n* = 850, CB: *n* = 144)			0.003			0.000
Very good/Good	217 (82.2)	525 (89.1)		9 (30.0)	98 (86.0)	
Not so good/Bad	47 (17.8)	61 (10.9)		21 (70.0)	16 (14.0)	
Working status (VB: *n* = 856, CB: *n* = 144)			0.048			0.000
Homemaker	203 (76.0)	409 (69.2)		7 (23.3)	72 (63.2)	
Earning own salary	64 (24.0)	180 (30.8)		23 (76.7)	42 (36.8)	
**Obstetric variables**						
Gravida (VB: *n* = 860, CB: *n* = 144)			0.295			0.516
Pregnant for first time	130 (48.3)	252 (42.6)		13 (43.3)	57 (50.0)	
Pregnant for second time	139 (51.7)	339 (57.4)		17 (56.7)	57 (50.0)	
Para (VB: *n* = 860, CB: *n* = 144)			0.105			0.218
Primiparous	138 (51.3)	268 (45.3)		12 (40.0)	60 (52.6)	
Multiparous	131 (48.7)	323 (54.7)		18 (60.0)	54 (47.4)	
Previous spontaneous abortions (VB: *n* = 860, CB: *n* = 144)			0.917			0.797
No	254 (94.4)	557 (94.2)		29 (96.7)	109 (99.0)	
Yes	15 (6.6)	34 (5.8)		1 (3.3)	1 (1.0)	
Period of gestation in current pregnancy (VB: *n* = 808, CB: *n* = 135)			0.710			0.910
Pre-term	32 (12.9)	62 (11.1)		1 (3.6)	6 (5.6)	
Term	175 (70.6)	409 (73.0)		21 (75.0)	79 (73.8)	
Late/Post-term	41 (16.5)	89 (15.9)		6 (21.4)	22 (20.6)	
**Intrapartum care-related variables**						
Level of health facility (VB: *n* = 851, CB: *n* = 140)			0.000			–
District Hospital	198 (74.4)	295 (50.4)		26 (100.0)	114 (100.0)	
Community Health Centre	68 (25.6)	290 (49.6)		0	0	
Time spent in the facility from arrival till childbirth (VB: *n* = 856, CB: *n* = 139)			0.138			0.750
≤6 hours	174 (60.7)	418 (70.7)		11 (39.3)	40 (36.0)	
>6 hours	91 (39.3)	173 (29.3)		17 (60.7)	71 (64.0(	
Had water to drink in early stages of labour (VB: *n* = 849)			0.308			
Yes	99 (11.7)	236 (27.8)		NA	NA	–
No	169 (19.9)	345 (40.6)		NA	NA	
Had light food in early stages of labour (VB: *n *= 844)			0.002			
Yes	82 (30.6)	241 (41.8)		NA	NA	–
No	186 (69.4)	335 (58.2)		NA	NA	
Hospital provided a sheet/blanket to cover herself inside the labour room/preparation room (VB *n* = 848, CB *n* = 144)			0.008			0.614
Yes	2 (7.5)	48 (8.3)		21 (70.0)	85 (74.6)	
No	266 (92.5)	532 (91.7)		9 (30.0)	29 (25.4)	
Had pillows to support herself on the labour cot/in preparation room (VB: *n* = 843, CB: *n* = NA)			0.263			0.982
Yes	178 (66.4)	359 (62.4)		NA	NA	
No	90 (33.6)	216 (37.6)		NA	NA	
Childbirth assisted by (VB: *n* = 848, CB: *n* = 144)			0.124			–
Physician/Obstetrician	3 (1.2)	17 (2.9)		30 (100.0)	114 (100.0)	
Nurse-Midwife/ANM	257 (98.8)	571 (97.1)		0	0	
Received perineal suturing for tear/episiotomy (VB: *n* = 859)						
Yes	122 (45.4)	232 (39.3)	0.096	NA	NA	–
No	147 (54.6)	358 (60.7)		NA	NA	
Received local anaesthesia before perineal suturing (VB: *n *= 353)						
Yes	39 (32.0)	106 (45.9)	0.011	NA	NA	–
No	83 (68.0)	125 (54.1)		NA	NA	
Sex of the newborn (VB: *n* = 860, CB: *n* = 144)			0.600			0.373
Female	125 (46.5)	286 (48.4)		18 (60.0)	58 (50.9)	
Male	144 (53.5)	305 (51.6)		12 (40.0)	56 (49.1)	
Weight of the newborn (VB: *n* = 829, CB: *n* = 134)			0.004			0.473
Extremely/Very low-birthweight babies (≤1500 g)	14 (5.4)	13 (2.3)		0 (0)	1 (0.9)	
Low-birthweight babies (1500–2500 g)	28 (10.8)	101 (17.7)		3 (10.0)	19 (16.7)	
Normal Indian weight range or above (≥2500 g)	216 (83.7)	457 (80.0)		27 (90.0)	84 (73.7)	
**Postpartum care related variables**						
Received own bed in the postnatal ward (VB: *n* = 855, CB: *n* = 144)			0.903			
Yes	266 (98.9)	580 (99.0)		30 (100.0)	114 (100.0)	–
No	3 (1.1)	6 (1.0)		0	0	–
Received a bedsheet from the hospital to cover the bed (VB: *n* = 854, CB: *n* = 144)			0.003			0.434
Yes	65 (24.2)	200 (34.2)		21 (70.0)	71 (62.3)	
No	204 (75.8)	385 (65.8)		9 (30.0)	43 (31.7)	
Received a bedside locker/place to store belongings during postnatal stay (VB: *n* = 850, CB: *n* = 143)			0.198			0.982
Yes	73 (27.1)	183 (31.5)		16 (53.3)	60 (53.1)	
No	196 (72.9)	398 (68.5)		14 (46.7)	53 (46.9)	
Received a bedsheet/blanket from the health facility to cover herself in postnatal ward (VB: *n* = 847, CB: *n* = 144)						0.751
Yes	18 (6.7)	194 (51.3)	0.008	12 (40.0)	42 (36.8)	
No	251 (93.3)	384 (66.4)		18 (60.0)	72 (63.2)	
Had privacy during breastfeeding the newborn (VB: *n* = 834, CB: *n* = 140)			0.000			0.336
Yes	58 (21.9)	311 (54.6)		13 (44.8)	39 (35.1)	
No	207 (78.1)	258 (45.3)		16 (55.2)	72 (64.9)	
Interaction with midwives for information and health education during hospital stay in early postpartum period (VB: *n* = 851, CB: *n *= 142)			0.000			0.492
No interaction at all once in postpartum ward	205 (77.4)	265 (45.2)		8 (27.6)	34 (30.1)	
Less than one interaction a day in postpartum ward	56 (21.1)	150 (25.6)		4 (13.8)	23 (20.4)	
One interaction a day or more in postpartum ward	8 (1.5)	171 (29.2)		17 (58.6)	56 (49.5)	
Monetary incentive for institutional births (VB: *n* = 860, CB: *n* = 144)			0.000			0.204
Received/will receive	203 (75.5)	506 (85.6)		21 (70.0)	92 (80.7)	
Not received/Do not know about it	66 (24.5)	85 (14.4)		9 (30.0)	22 (19.3)	
**Mental health-related variables**						
Fear of birth score (VB: *n* = 860, CB: *n* = 144)			0.030			0.000
Absent (WDEQ-B score <85)	260 (96.6)	584 (98.8)		14 (46.7)	8 (7.0)	
Present (WDEQ-B score ≥85)	9 (3.4)	7 (1.2)		16 (53.3)	106 (93.0)	
Depressive symptoms (VB: *n* = 860, CB: *n* = 144)			0.002			0.004
Absent	217 (80.7)	524 (88.7)		12 (40.0)	78 (68.4)	
Present	52 (19.3)	67 (11.3)		18 (60.0)	36 (31.6)	

ANM, Auxiliary Nurse and Midwife; CB, caesarean birth; SMMS, Scale for Measuring Maternal Satisfaction; VB, vaginal birth; WDEQ-B, Wijma Delivery Experience Questionnaire Version B.

Among women who had vaginal births, higher childbirth satisfaction also had an association with having given birth at a CHC (*p *< 0.01), having opportunity to interact with care providers at least once every day (*p *< 0.01), having a neonate with normal birthweight (*p *< 0.05), knowing that they had/would receive the monetary incentive given by the Government of India for institutional childbirths (*p *< 0.01), and being free from fear of childbirth (*p *< 0.05). Women who had vaginal childbirth were more satisfied when they received light food to eat (*p *< 0.05), received a sheet to cover themselves inside the labour room (*p *< 0.01), received bed linen (*p *< 0.05) and blankets (*p *< 0.05) for their stay in postnatal ward, and had privacy while breastfeeding their neonates (*p *< 0.01).

For caesarean births, the participants who experienced fear of childbirth were more satisfied with childbirth services compared to those women who were free of fear of childbirth (*p *< 0.05). The availability of the basic facilities mentioned above did not influence women’s satisfaction with caesarean births.

#### Regression analysis models for satisfaction with vaginal births and caesarean births

Socio-demographic factors, fear of birth, depressive symptoms and essential care-related factors were tested in one linear regression model (Table 5): age in years (continuous variable), perception of self-health, work status, weight of the newborn (continuous variable), fear of birth scores (continuous variable), depressive symptoms scores (continuous variable), privacy to breastfeed, perineal sutures received after birth, local anaesthesia received before suturing, and frequency of contact with nurse-midwives during hospital stay (continuous variable). Facility-related responses were tested in a separate regression model with overall SMMS–Normal Birth scores (Table 6): level of health facility, light food during early labour, cover-sheet inside labour room, bed linen in postnatal ward, blanket received in the postnatal ward, and monetary incentive for institutional childbirths.

**Table 5.** Linear regression model (satisfaction with vaginal births and sociodemographic, mental health and care-related factors).

	Coefficients^a^						
	Unstandardized coefficients		Standardized coefficients			95% CI for β	
Model	β	Std. Error	β	t	Sig.	Lower bound	Upper bound
(Constant)	159.915	18.011		8.879	0.000	124.478	145.352
Age <22 years	0.255	0.270	0.044	0.941	0.347	−0.277	0.787
Having positive perception of self-health	−2.300	2.558	−0.047	−0.899	0.369	−0.333	2.733
Earning own salary	−1.845	2.062	−0.044	−0.895	0.371	−1.902	2.211
Received perineal suturing due to tear or episiotomy	−.314	15.588	−0.001	−0.020	0.984	0.462	1.241
Received local anaesthesia before perineal wound suturing	−1.717	2.133	−0.046	−0.805	0.421	−5.914	2.479
Had opportunity to interact with nurse-midwives at least once a day	2.435	0.619	0.212	3.934	**0**.**000**	1.217	3.653
Gave birth to normal-weight neonates	−0.002	0.002	−0.075	−1.589	0.113	−0.005	0.001
Did not have depressive symptoms	−0.381	0.224	−0.101	−1.704	0.089	−0.821	0.059
Were free from the fear of childbirth	−0.267	0.073	−0.196	−3.675	**0**.**000**	−0.410	−0.124
Got privacy to breastfeed the newborn	−13.723	1.887	−0.371	−7.274	**0**.**000**	−12.435	−10.011

^a^Dependent Variable: SMMS–Vaginal Birth final total score.

**Table 6.** Linear regression model (satisfaction with vaginal births and basic facilities provided at the health facility).

	Coefficients^a^						
	Unstandardized coefficients		Standardized coefficients			95% CI for β	
Model	β	Std. error	β	t	Sig.	Lower bound	Upper bound
(Constant)	132.612	6.185		21.440	0.000	120.471	144.753
Gave birth at a CHC	8.791	1.261	0.244	6.973	**0**.**000**	6.316	11.265
Got light food to eat during early stage of labour	−3.008	1.275	−0.082	−2.359	**0**.**019**	−5.510	−0.505
Got a coversheet to cover herself in LR	−12.618	2.778	−0.169	−4.542	**0**.**000**	−18.071	−17.165
Got bedsheet from the hospital to cover the bed	−2.701	1.391	−0.070	−1.942	0.053	−5.433	0.030
Got a sheet from hospital to cover herself in ward	3.212	2.266	0.056	1.418	0.157	−1.236	7.661
Received monetary incentive for institutional birth	−1.963	1.612	−0.042	−1.218	0.224	−5.127	1.202

LR, labour room.

^a^Dependent variable: SMMS–Vaginal Birth final total score.

Women’s satisfaction with vaginal births had associations (Tables 5 and 6) with: (1) having given birth at a CHC (*p *< 0.001, 95% CI 6.316 to 11.265); (2) absence of fear of childbirth (*p *< 0.001, 95% CI 0.410 to −0.124); (3) light food in early labour (*p *< 0.05, 95% CI −5.510 to 0.505); (4) ability to maintain self-privacy inside the labour room (*p *< 0.001, 95% CI −18.071 to −17.165); (5) higher frequency of interactions with the nurse-midwives to seek information (*p *< 0.001, 95% CI 1.217 to 3.653); and (6) privacy during breastfeeding (*p *< 0.001, 95% CI −12.435 to −10.011), after adjusting for presence of depressive symptoms, age, work status, perception of self-health, neonatal birthweight, receiving perineal sutures, and receiving local anaesthesia for perineal suturing.

Factors having a significant association with overall SMMS–Caesarean Birth scores were tested as independent variables in linear regression analyses (Table 7): age in years (continuous variable), perception regarding self-health, work status, fear of birth scores (continuous variable), and depressive symptoms scores (continuous variable). After adjusting for women’s age, presence of fear of childbirth and presence of depressive symptoms, women’s satisfaction with caesarean birth was influenced by having positive perceptions towards self-health (*p *< 0.001, 95% CI −19.303 to −8.499) and earning their own salary (*p *< 0.001, 95% CI −15.171 to −5.680).

**Table 7.** Linear regression model (satisfaction with caesarean births).

	Coefficients^a^						
	Unstandardized coefficients		Standardized coefficients			95% CI for β	
Model	β	Std. error	β	t	Sig.	Lower bound	Upper bound
(Constant)	168.595	11.394		14.796	0.000	146.063	191.126
Age (years)	−0.213	0.361	−0.042	−0.589	0.557	−0.926	0.501
Positive perception of self-health	−13.901	2.732	−0.370	−5.089	**0**.**000**	−19.303	−8.499
Earning own salary	−10.425	2.400	−0.315	−4.344	**0**.**000**	−15.171	−5.680
Fear of childbirth	−0.031	0.063	−0.035	−0.496	0.621	−0.157	0.094
Depressive symptoms	−0.451	0.270	−0.120	−1.668	0.098	−0.986	0.084

^a^Dependent variable: SMMS–Caesarean Birth final total score.

## Discussion

Findings from this study reveal that while most of the women were satisfied with the childbirth services they received at the public health facilities, there were significant differences among women giving vaginal births at CHCs and at DHs. We interpret these findings in the light that while there is shortage of facilities at both CHCs and DHs (bed linen, cover, bedside locker, etc.), the heavy workload at the DHs further worsens the situation. CHCs, due to a lighter load of childbirths, are better able to keep the perinatal care wards prepared for women. While other Indian studies describe a shortage of infrastructure, material and supply to support increased numbers of institutional childbirths [39,40,56], our review did not reveal other quantitative studies to prove or disprove our interpretations.

On Hindi-SMMS, the mean subscale scores of women having vaginal births at the DH lay close the Likert scale median (score of 3). We interpret this findings in consideration of (1) the ‘Halo effect’ of giving birth influencing overall birth satisfaction, as has been mentioned in other studies [12,29]; (2) having very few expectations from institutional childbirth – namely the safety of self and the neonate, which were met by the health facility – gave the women birth satisfaction [34]; and (3) the women, in general, tended to respond with neutral or high scores on the satisfaction subscales, creating a ceiling effect. This tendency has been described in other studies too [34]. Among the whole group, the women who had caesarean births had a better likelihood of receiving basic facilities. We interpret this finding in light of the evidence that healthcare providers at the busy DHs prioritised the distribution of limited supplies to operative births to prevent infection and related complications.

Out of all the women who had perineal wounds due to vaginal childbirth – episiotomy and/or tears – one-third received perineal suturing without local anaesthesia. This malpractice was more commonly reported by the respondents at the DHs. Although this malpractice was not associated with level of satisfaction in this study, the process of surgical suturing without pain relief raises several ethical concerns. An impact of perineal wound suturing without local anaesthesia on women’s mental health – a substudy from the current cross-sectional survey – has been reported separately [53]. A review of Indian studies reveals only one qualitative study that mentions this malpractice [44]. However, other qualitative studies have reported that pain levels during labour and childbirth were considered to be ‘good’ by the Indian respondents [35,36,44]. To the best of our knowledge, no Indian study has yet explored the association between perineal wound suturing without local anaesthesia and birth satisfaction; however, our data show an association between perineal suturing without local anaesthesia and fear of childbirth [53], which in turn influences satisfaction with childbirth services. Interpersonal interactions with the nurse-midwives and personal privacy have strong associations with childbirth satisfaction in this study, and the positive influence of having the opportunity to interact with nurse-midwives (receiving information) and having privacy during the hospital stay is greater than the negative influence of not having proper infrastructure during the hospital stay. This finding is in line with other studies that have reported that processes surrounding childbirth have more influence on birth satisfaction compared with material aspects of care [4,5,29]. As others have reported, our findings show that having a fear of childbirth negatively influences the childbirth satisfaction of women who have vaginal births [57–59]. In this study, women who gave birth at CHCs were more satisfied with their birth experiences compared with those who gave birth at DHs. As CHCs tend to have significantly less childbirth-load, we interpreted that women in those facilities had better opportunities to receive care in a less crowded labour room with more attention given by the care providers. Studies from other countries also describe that crowded and busy labour rooms tend to have a negative impact on women’s birth experiences [60,61].

Similar to a recent Indian study that describes son-preference as a reason for large family size [62], in this study, the grandmultiparity was observed only among women who had only daughters. However, our study did not find any association between the sex of the neonate, or the proportion of sex among all living children, and birth satisfaction.

The Hindi-translated SMMS questionnaires were easy to administer, and required a short time to complete. Other studies are required to further test the applicability of using these questionnaires as a tool for the systematic improvement of services.

## Strengths and limitations of the study

To the best of our knowledge, this is the first Indian study to report an association between interpersonal interactions with care providers, and having privacy during the hospital stay with Indian women’s childbirth satisfaction while adjusting for fear of childbirth and depressive symptoms. The study was conducted over a large sample of women and the data were collected by an experienced research team following a rigorous guideline. Consecutive sampling among all public health facilities that had more than 10 childbirths in a month contributed towards preventing sample bias. Rigorous training of the research team on how to pose every item on the scale minimised potential bias. However, the short-term interpersonal relationship that developed between interviewer and interviewee could have created some response bias. Similarly, the findings present the expressed opinions of the respondents, and that may also create some response bias. This study has been conducted in two districts of Chhattisgarh, India, and the findings may not be applicable to every public health facility from every part of India. Also, this study has not included social indicators of satisfaction – such as marital relationship, addictions, and level of pain experienced in the postnatal period – in the analyses. Performing the data collection at the health facility within one week of childbirth may have created some response bias. Also, using standardised questionnaires in a clinical setting to measure a subjective phenomenon like satisfaction has limitations that the authors have tried to curtail, but which limit the interpretation due to the innate ‘rigidity’ associated with the questionnaires.

## Conclusion

Measuring women’s childbirth satisfaction is a complex multidimensional phenomenon that is influenced by women’s perceptions of quality care. However, women’s childbirth satisfaction provides crucial and cost-effective feedback for further improving institutional childbirth services. The current study shows that, while most of the women are satisfied with their childbirths, service gaps are visible across the childbirth and postnatal healthcare system: material and infrastructure shortage, delays in initiating maternal–neonatal bonding, and reduced opportunities for interpersonal interactions for most of the women. However, interpersonal interaction with care providers and providing privacy to the women during their childbirth and postpartum hospital stay have strong positive associations with women’s childbirth satisfaction. Immediate interventions to improve interaction and privacy are recommended to improve women’s birth satisfaction until the supply shortage is rectified.

## Paper context

Assessing quality of childbirth services has gained momentum in India, and several recent studies explore women’s satisfaction with their institutional births qualitatively. While the qualitative exploration yields in-depth information on childbirth satisfaction, having a short sample size confounds the transferability of findings to improve hands-on service. This quantitative study aimed to assess women’s satisfaction with childbirth care which, to a great extent, could be generalised and used for future interventions on improving women’s satisfaction with childbirth care.
